# Report of a Case of Spontaneous Rupture of the Uterus before Labour

**Published:** 1848-08

**Authors:** Thomas F. Brownbill

**Affiliations:** Surgeon to the Salford Workhouse


*Report of a case of Spontaneous Rupture of the Uterus before Labour.* By THOMAS F. BROWNBILL, ESQ., Surgeon to the Salford Workhouse.—M. A. G——, aged twenty-eight years, was of rather short stature, well proportioned, and had a healthy appearance. She had been married about eight years. In ten months subsequent to marriage, after an ordinary labour of about nine hours' duration, she gave birth to a full-grown female child, which lived about four months. Soon after labour, which I understand was quite natural, she was seized with convulsions, followed by delirium, &c., which, continuing for a week or ten days, subsequently resulted in an attack of puerperal mania, for which she was afterwards admitted into the Manchester Workhouse. Here she remained about two months, and, as no improvement had taken place, was then sent to Lancaster Asylum, whence, having been confined seven or eight months, she was discharged cured; and from that until a recent period had enjoyed uninterrupted good health. Having been separated from her husband during most of the time since her last confinement, she again became pregnant, and was admitted into the Salford Workhouse on the 4th of November last, in order to lie in. She stated that, in the beginning of the seventh month of gestation, whilst hanging out some clothes, she received a fall, which shook her violently, but did not cause her, either then or afterwards, any particular pain. On the 20th of November, at six A. M., after having passed a restless night, with occasional slight uterine pains, she began to vomit. This was followed by several pretty strong pains, during one of which she experienced (to use her own expression) a severe crack in the back, with a feeling of something suddenly giving way in her inside, which was immediately followed by a discharge of liquor amnii from the vagina. The midwife (an intelligent and experienced person) was accordingly sent for, and was soon in attendance. She found, upon examination, the os uteri nearly closed, hard, and incapable of admitting the point of the finger. There was a slight discharge, of a dark brown colour, from the vagina; the patient had vomited the contents of the stomach; and the pains had altogether subsided. Under these circumstances, she left her, and found, on her return, at three P. M., she had had no pain during her absence. The os uteri was lower down, and more yielding, though not in the least dilated, and a slight discharge of water, tinged with blood, escaped whilst making the examination. She had not slept or felt the motion of the child since soon after the membranes broke. A dose of castor oil was now ordered. On visiting her the following evening, at nine P. M., at the request of Mr. Roberts, the governor of the workhouse, I found the oil had been rejected by the stomach, and the vomiting had more or less continued to the present time, the matter at first being of a greenish yellow, and afterwards of a chocolate colour. Labour had not in the least progressed, the os uteri remaining as before, if anything, more contracted. She had no pains; complained of being weak and poorly; and although several opiates had at short intervals been administered, she had as yet not slept; and with a feeble pulse. Her countenance now began to assume an anxious expression.

Nov. 22d.—About eleven A. M. she began to doze for short periods, but this state soon gave way to extreme restlessness, almost incessantly requiring her position to be altered. She now complained of severe pain in the middle of her back, and her pulse was evidently sinking. Between one and two o'clock her breathing became laborious; her finger-nails had turned livid; continued gasping ensued; and in this state she died.

The body was inspected twenty-four hours after death, in the presence of several medical friends, and Mr. Roberts, the governor. The abdomen was found to contain a large quantity (about two pints) of dark-coloured, uncoagulated blood, probably diluted with a portion of the liquor amnii; and this being partially removed, the first object that presented itself, entirely excluded from the womb, and partially covered by the omentum and small intestines, was a full-grown male child, that had evidently been dead several days, the first stage of putrefaction having commenced. On partially removing the child, which lay with its left shoulder to the womb, a large rupture of this organ was observed, extending from the centre of the fundus, posteriorly, along its whole length as far as the os uteri, having only a narrow rim surrounding it, and through which the child had escaped into the cavity of the abdomen. The length of the opening was about seven inches, and the uterus, which seemed perfectly healthy, was well contracted over the firmly adherent placenta. In the above unfortunate case there are several points worthy of general notice, and which are of peculiar interest to the obstetrician. The patient was at the end of her calculation, and had a well-formed pelvis. The child was full-grown, of average size, also well-formed, and there existed between the two no disparity which would prevent the one easily passing through the other, supposing the presentation to be natural. From the time she first began to complain, up to her death, there was not the slightest pressure downwards; the os uteri was not at all dilated, and it firmly resisted the introduction of the finger-point, which effectually prevented my ascertaining the presentation of the child; nor was there any particular point or bulging perceptible in any part surrounding the os uteri, by which I could recognize its position. The The os uteri projected downward a little way into the vagina, and above it seemed to lead to an unobliterated cervix. Notwithstanding this state of things, from the total absence, since the first commencement, of anything like strong or bearing-down labour-pains, even at the time the waters escaped, it never occurred to me that the uterus had probably ruptured, which fact I first discovered at the post-mortem examination. The cause of the rupture is involved in much obscurity. She had not over-exerted herself, nor had she received any bodily injury since the time she fell; and the shake the fall occasioned was not followed by any soreness or other inconvenience. Towards the end of gestation she was often low-spirited, and entertained a presentiment, to which she often gave expression, that she should not survive the birth of her child. There was no softening of structure in the uterus, nor any indication of previous inflammation. The surrounding soft parts were healthy. The usual predisposing and exciting causes were all absent. Even supposing there were malposition of the fœtus, that the wall of the uterus should be endangered from such trivial pains seems surprising. The case altogether is remarkable, and it presents an instance of the least frequent form, as I believe, of this lesion—viz., the longitudinal rupture of this organ, extending from the centre of the fundus, posteriorly, in a straight direction, to within half an inch of the posterior margin of the os uteri.—*Lancet.*

